# Plantar Erythrodysesthesia Caused by Antiretroviral Treatment: A Case Report and Review of the Literature

**DOI:** 10.1155/2013/757496

**Published:** 2013-06-26

**Authors:** B. Aigner, K. Brockow, U. Darsow, J. Ring, S. G. Plötz

**Affiliations:** ^1^Dermatology Munich-Harlaching, Grünwalderstraße 248, 81545 Munich, Germany; ^2^University Clinic of Dermatology and Venerology, Medical University Graz, Auenbruggerplatz 8, 8036 Graz, Austria; ^3^Department of Dermatology and Allergy am Biederstein, Technical University Munich, Biedersteinerstraße 29, 80802 Munich, Germany; ^4^Christine Kühne-Center for Allergy Research and Education (CK-CARE), Technical University Munich, Biedersteinerstraße 29, 80802 Munich, Germany

## Abstract

Palmoplantar erythrodysesthesia is an uncommon localised cutaneous reaction to certain chemotherapeutic agents and characterized by painful palmoplantar erythema and dysesthesia. To the best of our knowledge, we report the first case of plantar erythrodysesthesia in a 40-year-old male patient receiving an antiretroviral combination therapy for HIV.

## 1. Introduction 

Palmoplantar erythrodysesthesia (PPE), known as hand-foot syndrome is an uncommon, distinct, localised cutaneous reaction in patients treated with high dose chemotherapy [1, 2]. It is characterized by symmetrical palmoplantar erythema, edema, dysesthesia, and a variety of clinical symptoms. More than a dozen of drugs have been implicated, with high dose traditional chemotherapeutic agents and kinase inhibitors like sorafenib and sunitinib [1, 2]. Lopinavir and ritonavir, two protease inhibitors, are used for antiretroviral therapy in HIV infected patients. While most frequently reported adverse effects caused gastrointestinal symptoms, cutaneous reactions have been seldom described [3–5]. Emtricitabine and tenofovir are both nucleoside analogue HIV-1 reverse transcriptase inhibitors and generally well tolerated. However cutaneous reactions have been described using these drugs [4, 5]. Up till today, no occurrence of plantar erythrodysesthesia in a patient receiving lopinavir, ritonavir, emtricitabine, and tenofovir has been described in the literature. 

## 2. Case Presentation

**Figure 1:**
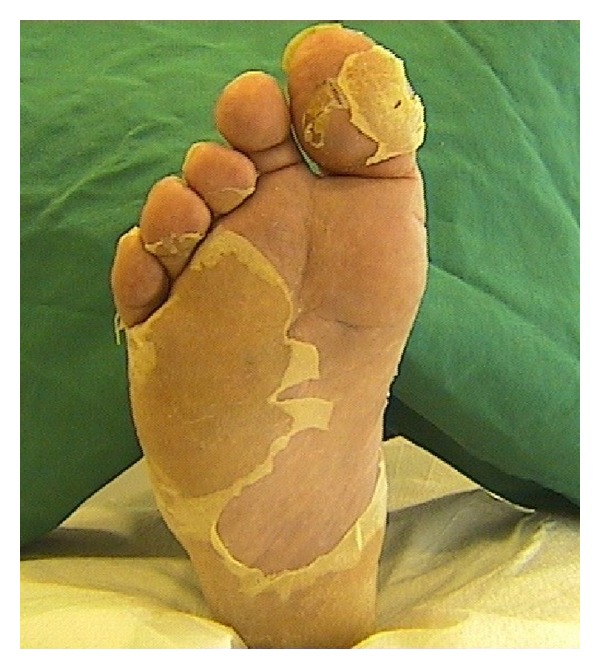
Erythematous desquamation of the right planta pedis, of a 40-year old patient who was treated with an antiretroviral combination therapy and developed plantar erythrodysesthesia.

A 40-year-old Caucasian male, who had been diagnosed for HIV infection in 2004, presented with plantar erythema, dysesthesia, pruritus, and desquamation of the skin. The symptoms were recurrent with varying severity. Upon dermatological examination his soles displayed erythematous macules and exfoliation, affecting the whole plantar region (Figure 1). His peripheral neurological status was inconspicuous. Medication at time of diagnosis consisted in antiretroviral combination therapy, including lopinavir and ritonavir (2-0-2) as well as emtricitabine and tenofovir (1-0-0). The patient reported previous episodes of erythema and desquamation of the plantar region but denied any other drug intolerance, other hypersensitivity reaction, or drug abuse. He had a medical history of alcohol abuse but no further morbidities. Laboratory evaluation revealed the patient's chemistry profile and blood count within normal values except the following elevated liver enzymes: GOT 63 U/L, GPT 73 U/L, and GGT 162 U/L). Hepatitis B and C antibodies were found to be negative. The patient denied biopsy of the plantar lesions and mycological cultures were negative. To avoid interruption of essential treatment, we applied topical photochemotherapy cream (PUVA) up to a dose of 3 joules. Skin lesions improved gradually. The patient did not appear to the proposed allergy diagnostic work-up.

## 3. Discussion

We report the case of repetitive occurrence of plantar erythema, dysesthesia, pruritus, and desquamation in a 40-year-old male caucasian patient with HIV infection and antiretroviral combination therapy.

The pathophysiology of PPE is still unknown and several mechanisms have been proposed [1, 2]. It has been suggested that PPE is caused by extravasation of the drug from palmoplantar microcapillaries due to local traumas of daily activities on mechanically stressed skin sites.

Dose reduction or interruption of medication is the only causal evaluated treatment options in PPE described so far [2]. Moreover, vitamin B6 (pyridoxine), cyclooxygenase-2-inhibitors (COX 2), and vasoconstrictive therapy, like cooling acral areas, have been investigated and proven beneficial [6]. In addition, appropriate analgesia is crucial. The authors used PUVA, which is a fast, effective, and easily applicable treatment especially in palmoplantar skin lesions. 

Especially in patients suffering from HIV, the identification of a causative single antiretroviral drug is complicated, because most patients are treated with combined therapies and discontinuation is not possible. Cutaneous adverse effects are not uncommon [5]. However in most patients systemic adverse events are predominant. Patients exposed to tenofovir have to be monitored for renal insufficiency and osteoporosis due to its metabolism [5]. The intake of protease inhibitors, like lopinavir and ritonavir, can lead to a variety of systemic symptoms such as dysmetabolism, but cutaneous and allergic reactions have been described as well [4]. However, in the presented case, an allergological work up could not be performed due to the low adherence to appointments in this patient who missed allergological exams and further laboratory examinations. The authors believe that allergological exams are mandatory in such cases to differentiate allergological from toxic effects of the administered medication. In general, cutaneous side effects are rare [5]. However, in 2001 Lascaux et al. reported about cutaneous drug reactions due to lopinavir, as an inflammatory oedema of the legs could be observed [7]. Further cutaneous drug reactions due to antiretroviral therapy have been reported in 2003 [8], as well as the occurrence of macula-papular rashes in patients who received lopinavir and ritonavir and developed a severe, itchy macula-papular drug reaction [3]. Emtricitabine can lead to xerosis cutis, rash, pruritus, urticaria, and self-limited vesiculobullous disease [5]. Interestingly, emtricitabine is known to elicit hyperpigmentation of the palms or soles [5]. In 1993 Pedailles et al. were the first to describe PPE in a patient who was diagnosed for HIV and had been treated with a reverse transcriptase inhibitor (didanosine) [9]. Although didanosine is believed to elicit only few skin reactions, in single cases it could be associated with severe cutaneous reactions. 

As cutaneous reactions due to ritonavir, lopinavir, and tenofovir are rare [5] and the only described case of PPE connected to antiretroviral therapy has been associated with a reverse transcriptase inhibitor, we hypothesize that emtricitabine is the elicitor of PPE in our patient. 

## 4. Conclusion

Patients receiving antiretroviral drugs frequently experience cutaneous side effects. To avoid treatment interruptions of life-saving medication, early recognition, understanding of pathogenesis, and adequate therapy for this predominantly dose limited syndrome are crucial. We recommend greater vigilance for palmoplantar erythrodysesthesia in patients, treated with lopinavir, ritonavir, emtricitabine, and tenofovir.
